# Comparative Analysis of Amaryllidaceae Alkaloids from Three *Lycoris* Species

**DOI:** 10.3390/molecules201219806

**Published:** 2015-12-07

**Authors:** Yongqiang Tian, Chunyun Zhang, Mingquan Guo

**Affiliations:** 1 Sino-Africa Joint Research Center/Key Laboratory of Plant Germplasm Enhancement and Specialty Agriculture, Wuhan Botanical Garden, Chinese Academy of Sciences, Wuhan 430074, China; xiaoqiangtian2013@163.com (Y.T.); cyzhang@wbgcas.cn (C.Z.); 2 Graduate University of Chinese Academy of Sciences, Beijing 100049, China

**Keywords:** Amaryllidaceae alkaloids, chemical fingerprints, chemical marker, anti-HepG2 activity

## Abstract

The major active constituents from Amaryllidaceae family were reported to be Amaryllidaceae alkaloids (AAs), which exhibited a wide spectrum of biological activities, such as anti-tumor, anti-viral, and acetyl-cholinesterase-inhibitory activities. In order to better understand their potential as a source of bioactive AAs and the phytochemical variations among three different species of *Lycoris* herbs, the HPLC fingerprint profiles of *Lycoris* *aurea* (*L. aurea*), *L. radiata*, and *L. guangxiensis* were firstly determined and compared using LC-UV and LC-MS/MS. As a result, 39 peaks were resolved and identified as AAs, of which nine peaks were found in common for all these three species, while the other 30 peaks could be revealed as characteristic AAs for *L. aurea*, *L. radiata* and *L. guangxiensis*, respectively. Thus, these AAs can be used as chemical markers for the identification and quality control of these plant species. To further reveal correlations between chemical components and their pharmaceutical activities of these species at the molecular level, the bioactivities of the total AAs from the three plant species were also tested against HepG2 cells with the inhibitory rate at 78.02%, 84.91% and 66.81% for *L. aurea*, *L. radiata* and *L. guangxiensis*, respectively. This study firstly revealed that the three species under investigation were different not only in the types of AAs, but also in their contents, and both contributed to their pharmacological distinctions. To the best of our knowledge, the current research provides the most detailed phytochemical profiles of AAs in these species, and offers valuable information for future valuation and exploitation of these medicinal plants.

## 1. Introduction

Belonging to the Amaryllidaceae family, plants of genera *Lycoris* were known not only for their ornamental value, but also for their medicinal value being used as a medicinal herb for thousands of years in China [1,2]. It has been well documented that the Amaryllidaceae alkaloids (AAs) were responsible for their pharmaceutical activities, and exhibited a wide spectrum of biological activities, such as anti-tumor, anti-malarial, and acetylcholinesterase inhibitory activities [3,4,5,6,7,8]. In turn, there has been growing interest in the search for new AAs with better bioactivities from Amaryllidaceae plants [9]. In the past few years, more and more alkaloids were isolated from the Amaryllidaceae family, and most of which belong to galanthamine type, lycorine type, homolycorine type, tazettine type and crinine type in terms of chemical structures [10]. Among them, galanthamine and lycoramine were reported to exhibit good activity against Alzheimer’s disease [7]. While more AAs, such as lycorine, dihydrolycorine, haemanthamine, pretazettine, pseudolycorine, and narciclasine, showed significant activity against a variety of cancer cells either by inhibiting cancer cell growth mainly through cytostatic effects targeting small RHO GTPases or through the inhibition of protein synthesis and the subsequent disorganization of the actin cytoskeleton [11,12,13,14]. Due to the remarkable pharmaceutical activities, AAs have led to increasing interest in the search for new resources and new bioactive components from different species in the Amaryllidaceae family. However, most of the current research only focused on certain major species on the market, and little work has been conducted for the comprehensive analysis of AAs from different *Lycoris* species. Since remarkable chemical differences have often been found in different species of medicinal plants or even from different geographic origins, which significantly affected quality and bioactivities. In most cases, the chemical differences often resulted in pharmacological distinctions. In this context, we set out to investigate and compare chemical fingerprint profiles of three *Lycoris* species. Due to the complexity and variety of components in these plant species, it is of primary importance to develop a tailored analytical method for the comprehensive analysis of AAs from these medicinal plants [15,16].

In the past few decades, a number of analytical methods including GC-MS, LC-MS, and CE-ESI-IT-MS have been developed for the analysis of AAs [17,18,19,20,21,22,23], which contributed significantly to the better understanding of AAs from medicinal plants. Due to the high sensitivity and the access to the MS database, GC-MS was deemed as an effective method for the analysis of AAs in the past [19]. However, it was limited to AAs with relatively higher volatility, which was inappropriate to some AAs, especially in this work. Compared to GC-MS, LC-MS has been more widely used in the analysis of alkaloids in various plant sources due to its ability in detecting thermo-unstable and high-molecular-weight alkaloids in recent years [19,21], and it is thus applied in this study. While used for different research purposes, those LC-MS methods reported have limitations in both their resolution and capacity of profiling AAs, and can only analyze one or a very limited number of AAs [23,24,25,26,27]. In order to conduct a comprehensive analysis of AAs, and overcome these limitations, a more effective method is required to compare fingerprint profiles of different *Lycoris* species. Thus, a rapid, sensitive, and reliable HPLC-UV/ESI-MS/MS method has been successfully developed for the comparative analysis of the AAs from different *Lycoris* species, which resulted in the simultaneous separation and identification of over 30 AAs from different *Lycoris* species under the optimized conditions. To the best of our knowledge, the present study is the first report on qualitative and quantitative assessment of AAs from different *Lycoris* species, and provides an important clue for future valuation and exploitation of these medicinal plants.

## 2. Results and Discussion

### 2.1. Optimization of Chromatographic Conditions

Previous phytochemical investigations into AAs in *Lycoris* species revealed its complexity in chemical structure and the diversity in AAs types, e.g., galanthamine, galanthine, lycorine, lycoramine, narwedine, tazettine, and haemanthamine [17,18]. This posed significant challenges to the comprehensive analysis of AAs from different species. Considering the structural differences of AAs and other reported LC methods, chromatographic conditions, such as wavelength of maximum absorption, LC columns, mobile phase and mobile phase additives, were systematically investigated to achieve better separation of AAs. By scanning the samples extracted from different species, the maximum absorption wavelength at 232 nm was selected for LC-UV analysis in this study. Based on the column efficiency, and the resultant chromatograms, Phenomenex ODS column (150 × 2.00 mm, 5 μm, Phenomenex, Torrance, CA, USA) was chosen as a preferred one over the reverse phase C18 column (Sunfire C18, 150 mm × 4.6 mm, 3.5 μm, Waters, Milford, MA, USA). By stepwise investigation of mobile phase and additives, including methanol-water, acetonitrile-water, acetonitrile-0.1% formic acid, acetonitrile-10 mM ammonium acetate, and acetonitrile-40 mM ammonium acetate, the combination of acetonitrile and 40 mM ammonium acetate was chosen as the mobile phase and additives according to their better performance and resolution in the gradient elution at 232 nm, respectively. The final optimized conditions for the analysis of AAs from different *Lycoris* species are given in Section 3.3.1.

### 2.2. Sample Analysis and Amaryllidaceae Alkaloids’ (AAs) Identification

#### 2.2.1. HPLC Fingerprint Profiles

**Figure 1:**
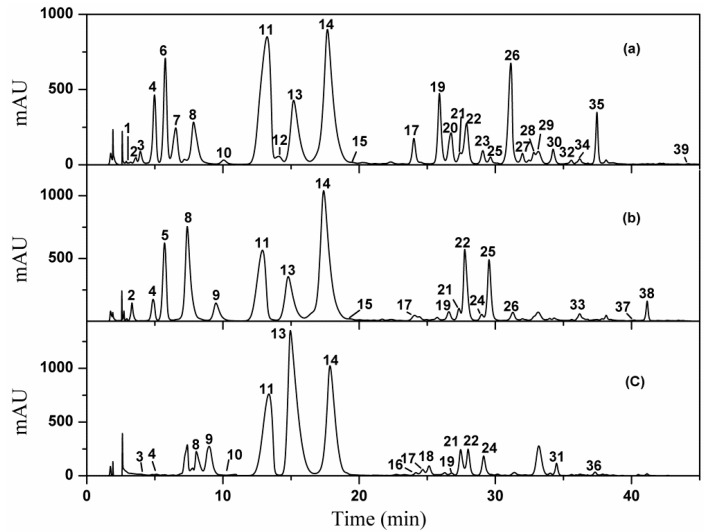
HPLC profiles of *L. radiata* (**a**); *L. aurea* (**b**) and *L. guangxiensis* (**c**).

Total AAs extracted from the bulks of the three Lycoris species were further enriched by Oasis MCX SPE cartridges, and subsequently analyzed in parallel on a Phenomenex column under the optimized LC conditions. As shown in Figure 1a–c, the HPLC fingerprinting profiles of AAs from the three species were determined. It was observed that 39 peaks were well resolved under the given HPLC conditions, indicating that the optimized conditions were achieved for the comparative analysis of AAs from the three Lycoris species.

#### 2.2.2. Identification of AAs

**Figure 2:**
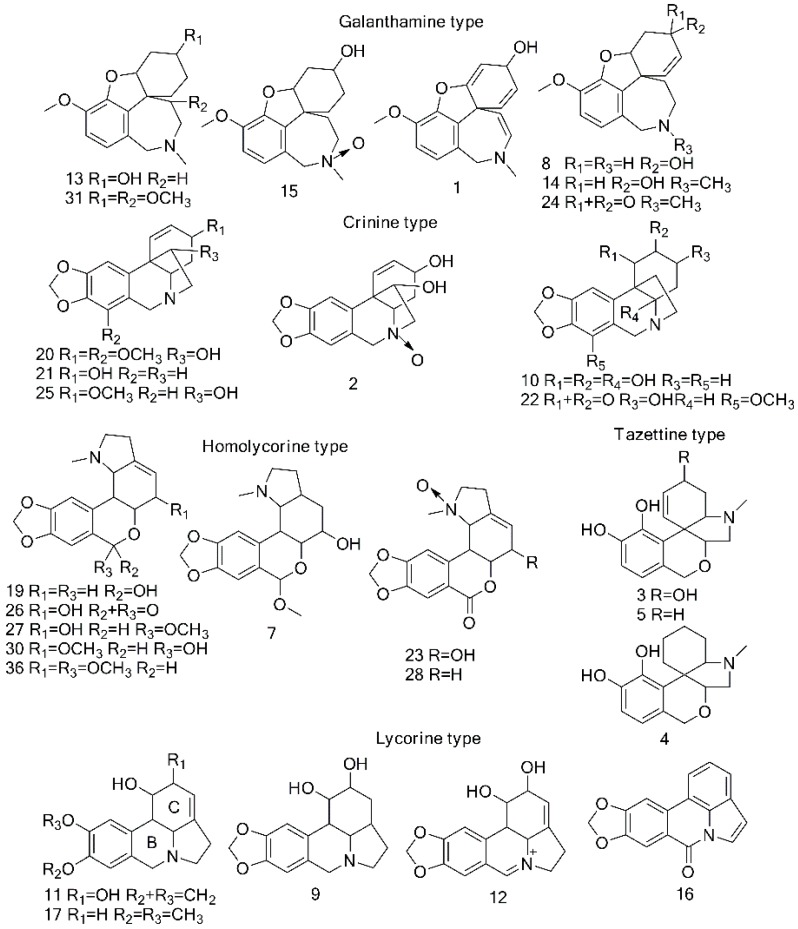
Chemical structures of the AAs identified from *L. aurea*, *L. radiata* and *L. guangxiensis* (Note: the numbers correspond to the peak numbers shown in Figure 1).

According to the HPLC profiles (Figure 1), 39 AAs were detected from the three *Lycoris* species, which were listed in Table 1. Among them, 29 AAs were identified in this study by comparing their MS/MS with the corresponding authentic standards or published literatures as shown in Table 1. To the best of our knowledge, seven of the AAs (labeled as “a” in Table 1) were firstly reported in genera *Lycoris*, and five of them (labeled as “b” in Table 1) were firstly reported in the Amaryllidaceae family. Based on the identifications, the chemical structures of the 29 AAs were summarized in Figure 2. Since different types of AAs displayed different fragment pathways using ESI-MS/MS, the structural diversity of AAs made their identification very complicated. To simplify the interpretation of the obtained MS/MS data for AAs, we classified them into five groups according to their chemical structures, *i.e.*, galanthamine type, lycorine type, crinine type, homolycorine type and tazettine type [17,24,25]. In more detail, the representative interpretations of the MS/MS data for five alkaloid types were discussed as below.

**Table 1:** Peaks detected as AAs by LC-MS/MS.

Peak No.	R_t_ (min)	PI-MS [M + H]^+^	MS/MS Data	Identification	Alkaloid Type/Ref.
1	3.22	284	266, 256, 249, 241, 237, 226, 213, 199	1,11-didehydrogalanthamine ^b^	GA [17]
2	3.61	304	286, 268, 250, 227, 211, 150	11-hydroxyvittatine N-oxide ^a^	CR [27,33]
3	3.93	290	272, 225, 217, 211, 201, 199, 181, 124, 119	3-hydroxylatifaliumin C ^b^	TA [33]
4	4.97	276	258, 219, 215, 201, 189, 175, 153	Dihydro-latifaliuminC ^a^	TA [33]
5	5.71	274	256, 225, 217, 207, 199, 197, 181	Latifaliumin C ^a^	TA [33]
6	5.76	350	332, 281, 267, 255, 223, 193, 180	Unidentified	
7	6.54	334	316, 298, 270, 267, 255, 238, 173	2α-Hydroxy-3-hydro-6-*O*-methyloduline ^b^	HO [36]
8	7.17	274	231, 225, 213, 198, 183	N-demethyl-galanthamine	GA [26,27]
9	9.48	290	272, 254, 226, 149, 136, 112, 68	Dihydrolycorine	LY [29,30,31]
10	10.05	306	288, 270, 229, 189	Crinamabine ^a^	CR [35]
11	12.90	288	270, 252, 222, 177, 147, 119, 95	Lycorine	LY [1,17]
12	14.08	286	250, 240, 226, 147	(+)-5,6-dehydrolycorine	LY [5]
13	14.79	290	272, 233, 215, 189	lycoramine	GA [1]
14	17.40	288	270, 231, 225, 213, 198, 181	Galanthamine	GA [17]
15	18.24	306	288, 247, 233, 229, 215, 201, 189	lycoramine N-oxide	GA [15,25]
16	22.04	264	247, 189, 166, 149, 133, 116	Hippadine	LY [32]
17	23.23	288	270, 255, 239, 193, 162, 151, 121, 108, 94	Pluviine	LY [16,19]
18	24.20	262	244, 228, 219, 205, 179, 165, 147, 123, 98, 91	Unidentified	
19	26.58	302	284, 266, 255, 193, 175, 145, 108, 94	Oduline	HO [36]
20	26.74	332	300, 282, 264, 234, 225, 213, 199, 169	Ambelline ^a^	CR [17,34]
21	27.33	272	254, 242, 226, 149, 136, 108	Vittatine	CR [17]
22	27.88	318	300, 286, 268, 250, 227, 209, 199, 149	Crinamidine ^a^	CR [17,33]
23	29.08	332	300, 282, 275, 267, 255, 243, 223, 195, 124	Hippeastrine N-oxide	HO [37]
24	29.15	286	255, 229, 225, 197, 179, 168, 58	Narwedine ^a^	GA [28]
25	29.64	302	270, 259, 226, 211, 196, 181, 168	Haemanthamine	CR [17]
26	30.96	316	298, 280, 273, 239, 222, 191 ,126, 96, 83	hippeastrine	HO [36]
27	31.86	332	314, 282, 253, 239, 211, 223, 175, 96	2α-Hydroxy-6-*O*-methyloduline	HO [36]
28	32.82	316	298, 280, 267, 239, 207, 191, 176, 160, 108, 94	(+)-8,9-methylenedioxylhomolycorine N-oxide	HO [5]
29	33.15	318	286, 271, 267, 177	Unidentified	
30	33.38	332	300, 282, 257, 251, 243, 191, 163, 94,	2-methoxyoduline ^b^	HO [36]
31	34.49	334	302, 270, 259, 245, 231, 217, 213, 199	3,11-dimethoxy-lycoramine ^b^	GA [17]
32	35.58	316	285, 267, 256, 239, 228, 207, 175, 157, 129, 118	Unidentified	
33	36.19	332	300, 284, 271, 251, 239, 219, 191, 94, 81	Unidentified	
34	36.19	266	250, 236, 222, 208, 109	Unidentified	
35	36.84	332	314, 300, 282, 271, 264, 240, 224, 211, 181, 153, 120, 107	Unidentified	
36	37.56	346	314, 282, 253, 239, 225, 211, 175, 147, 96	2α-Methoxy-6-*O*-methyloduline	HO [36]
37	40.20	344	312, 280, 266, 252, 195, 89	Unidentified	
38	41.15	298	270, 248, 238, 212, 180	Unidentified	
39	44.57	346	288, 241, 239, 211, 209, 183, 168, 140, 116, 94	Unidentified	

Abbreviations: GA, galanthamine type; CR, crinine type; TA, tazettine type; HO, homolycorine type; LY, lycorine type; ^a^: firstly reported in lycoris genera; ^b^: firstly reported in Amaryllidaceae family.

#### 2.2.3. Identification of Galanthamine Type Alkaloids

For the identification of galanthamine type alkaloids, the loss of N-methyl and vicinal carbon atoms was the typically characteristic fragmentation pathways in the MS analysis among AAs. According to the MS/MS spectra, seven peaks, numbered as **1**, **8**, **13**, **14**, **15**, **24**, and **31**, were identified and classified into this group.

By comparing the MS/MS data with the authentic standard, peaks **13** and **14** were definitely identified as lycoramine and galanthamine, respectively [1,17]. For lycoramine (peak **13**), several abundant fragment ions at *m*/*z* 272 [M + H − H_2_O]^+^, 233 [M + H − C_3_H_7_N]^+^, and 215 [M + H − H_2_O − C_3_H_7_N]^+^ were observed, and another fragment ion at *m*/*z* 189 was attributed to the further loss C_2_H_2_ (26 Da) from fragment ion at *m*/*z* 215. For galanthamine (peak **14**), the fragment ion at *m*/*z* 270 was formed by the neutral loss of H_2_O. The further loss of the N vicinal moiety from fragment ion at *m/z* 270 produced fragment ions at *m*/*z* 231, 225, 213 and 198. The fragment ion at *m*/*z* 181 was observed due to the further neutral loss of CH_3_OH (32 Da) from ion at *m*/*z* 213.

**Figure 3:**
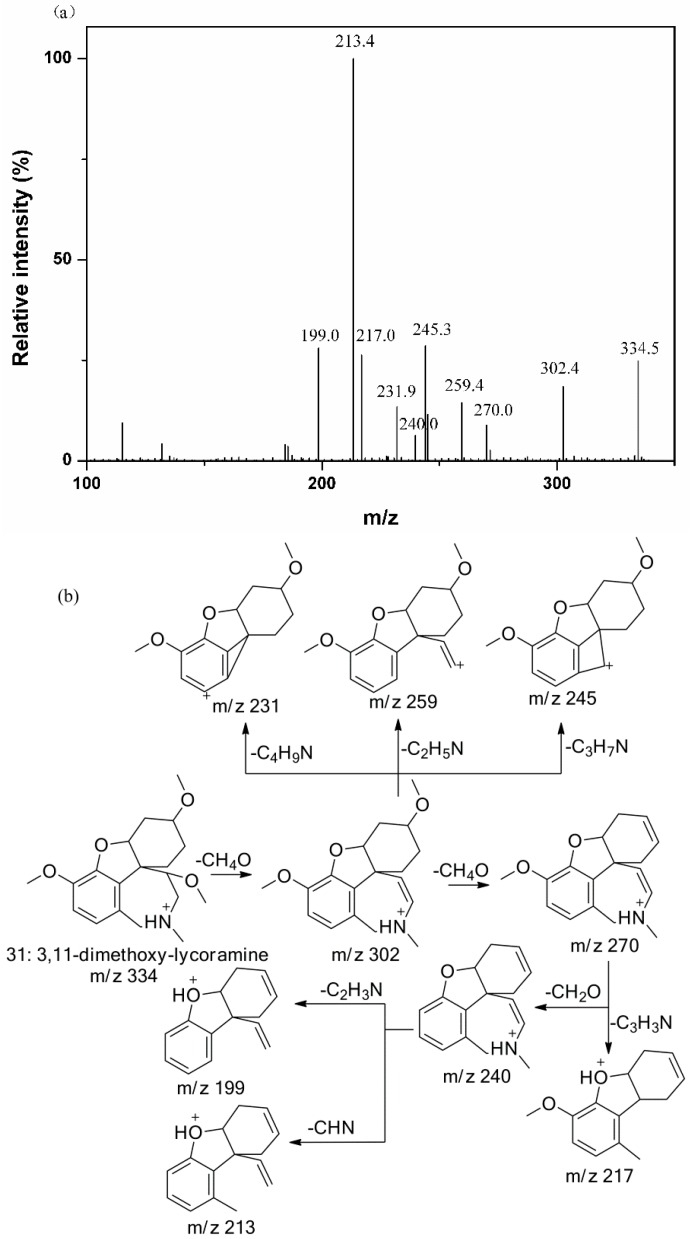
The MS/MS spectrum of peak 31 (**a**) and the proposed fragmentation pathways (**b**).

Figure 3 showed the MS/MS spectrum and the fragmentation pathways of peak **31**, in which several abundant fragments at *m*/*z* 231, 213 and 198 were found very similar to galanthamine. Since peak **31** showed a molecular ion at *m*/*z* 334 ([M + H]^+^) and two subsequent fragment ions at *m*/*z* 302 ([M + H − CH_3_OH]^+^) and at *m*/*z* 270 ([M + H − 2CH_3_OH]^+^), suggesting the presence of two methoxy groups, thus it can be tentatively identified as 3, 11-dimethoxy-lycoramine [17]. Similar to MS/MS of galanthamine, several typical fragment ions at *m*/*z* 231, 213 and 198 were also observed for peak **8**. Considering the molecular ion of peak **8** at *m*/*z* 274 [M + H]^+^ (C_16_H_19_NO_3_), peak **8** was thus tentatively identified as *N*-demethyl-galanthamine [26,27]. In this way, peak **1** could be tentatively identified as 1,11-didehydrogalanthamine, since its molecular ion at *m*/*z* 284 [M + H]^+^ (C_19_H_27_NO_4_), and similar fragment ions at *m*/*z* 213 and 198 to those of galanthamine were observed along with an abundant fragment ion at *m*/*z* 266 [17].

In addition, peak **15** was also tentatively identified as lycoramine N-oxide based on the characteristic fragment ions at *m*/*z* 233, 215 and 189 similar to galanthamine and its molecular ion observed at *m*/*z* 306 ([M + H]^+^), along with some weak fragment ions at *m*/*z* 288 and 247 by the successive loss of H_2_O (18 Da) and C_3_H_3_N (53 Da) [15,25,26]. As for peak **24**, a neutral loss of CH_3_NH_2_ (31 Da) from the molecular ion at *m*/*z* 286 [M + H]^+^ resulted in a fragment ion at *m*/*z* 255, and the fragment ions at *m*/*z* 225, 197 and 179 could be attributed to the successive loss of CH_2_O, CO and H_2_O from fragment ion at *m/z* 255. Another abundant fragment ion at *m*/*z* 229 [M + H − C_3_H_7_N]^+^ observed was probably produced by the loss of N moiety C_3_H_7_N (58 Da).Thus, peak **24** was tentatively identified as narwedine [28].

#### 2.2.4. Identification of Lycorine Type Alkaloids

Through the investigation on fragment pathways of lycorine type alkaloids, the unique structures of this type typically produced characteristic fragment ions via Retro-Diels-Alder (RDA) cleavages. Based on their MS/MS data, peaks **9**, **11**, **12**, **16**, and **17** were identified and classified into lycorine type alkaloids.

Among the lycorine type alkaloids identified, both peak **11** and peak **17** exhibited a [M + H]^+^ ion at *m*/*z* 288. For peak **11**, in addition to the RDA cleavages of B and C rings produced two characteristic fragment ions at *m*/*z* 177 [M + H − C_6_H_9_NO]^+^ and 147 [M + H − C_7_H_11_NO_2_]^+^, the successive loss of H_2_O, 2H_2_O and CH_2_O from the parent ion at *m*/*z* 288 yielded three fragment ions at *m*/*z* 270, 252 and 222, respectively. In reference to the MS/MS data from the authentic standard [1,17], peak **11** was identified as lycorine. As for peak **17**, the loss of C_6_H_9_N (95) and C_11_H_14_O_3_ (194) from [M + H]^+^ led to the characteristic fragment ions at *m*/*z* 193 and 94 by RDA cleavages and the fragment ion at *m*/*z* 270 was observed due to the loss of H_2_O (18 Da). The further RDA cleavage of fragment at *m*/*z* 270 yielded two fragment ions at *m*/*z* 255 [M + H − H_2_O − NH]^+^ and *m*/*z* 239 [M + H − H_2_O − CH_5_N]^+^. Thus, peak **17** was finally identified as pluviine [16,29]. In this way, peak **9** was also identified based on the typical fragment ions at *m*/*z* 272, 254 and 149, which were 2 Da more than those corresponding ions at *m*/*z* 270, 252 and 147 for lycorine. Therefore, peak **9** was identified as dihydrolycorine [26,29,30,31]. For peak **12**, the loss of H_2_O and C_2_H_2_O from the molecular ion at *m*/*z* 286 [M + H]^+^ produced abundant fragment ions at *m*/*z* 250 [M + H − 2H_2_O]^+^ and 226 [M + H − H_2_O − C_2_H_2_O]^+^ by successive neutral losses. Meanwhile, the fragment ion at *m/z* 147 was also observed by the loss of C_7_H_7_NO (121 Da) due to RDA cleavages. Thus, peak **12** was tentatively identified as (+)-5,6-dehydrolycorine [5]. In respect to peak **16**, several abundant fragment ions at *m*/*z* 247, 149 and 166 were produced mainly via RDA cleavages. As a result, it was tentatively identified as (+)-5,6-dehydrolycorine based on the fragment pathways above [32].

#### 2.2.5. Identification of Crinine Type Alkaloids

In terms of MS fragmentation pathways of crinine type alkaloids, the abundant fragment ions observed in the MS/MS spectrum were produced due to both RDA and α-cleavages. As shown in Table 1, peaks **2**, **10**, **20**, **21**, **22** and **25** were classified into crinine type group based on their characteristic fragment ions.

Compared the MS/MS data with those from authentic standards, peak **21** and **25** were identified as vittatine and haemanthamine [17]. For peak **21**, except for the most intensive and characteristic fragmention at *m*/*z* 136 produced due to the RDA and α-cleavages, the fragment ions at *m*/*z* 254, 240, and 226 were observed by successive neutral losses from the molecular ion at *m*/*z* 272 [M + H]^+^. As for peak **25**, the characteristic fragment ion at *m*/*z* 259 [M + H − 43]^+^ was produced by RDA and α-cleavages, and the fragment ion at *m*/*z* 226 was observed due to the neutral loss of CH_3_OH and further dissociation of an H radical from the fragment ion at *m*/*z* 259. Fragment ions at *m*/*z* 270, 252, 211 and 181 were generated by the successive neutral loss of CH_3_OH (32 Da) , H_2_O (18 Da), as well as RDA cleavage radical eliminating C_2_H_3_N (41 Da) and CH_2_O (30 Da) from the molecular ion at *m*/*z* 302 [M + H]^+^.

In regards to peaks **2** and **22**, the similar fragment ions at 286, 268, 250 and 227 were observed. Peak **2** showed a molecular ion at *m*/*z* 304 [M + H]^+^, and a differential fragment ion at *m*/*z* 150 was probably generated by the loss of C_7_H_2_O_2_ from the fragment ion at *m*/*z* 268. For peak **22**, the loss of H_2_O from the molecular ion [M + H]^+^ at *m*/*z* 318 produced the fragment ion at *m*/*z* 300, and then a further loss of H_2_O and CO from fragment ion at *m*/*z* 227 resulted in the fragment ions at *m*/*z* 209 and 199, respectively. Based on interpretations of these fragment ions above, peaks **2** and **22** could be tentatively identified as 11-hydroxyvittatine N-oxide and crinamidine, respectively [17,27,33]. With the molecular ion at *m*/*z* 332 ([M + H]^+^), peak **20** exhibited the similar MS/MS spectrum as that of peak **22** and was identified as ambelline [3,17]. As for peak **10**, with the molecular ion at *m*/*z* 306 ([M + H]^+^), its four major fragment ions at *m*/*z* 288, 270, 229 and 152 were 2 Da more than those corresponding fragment ions at *m*/*z* 286, 268, 227 and 150 from peak **2**, respectively, along with another two differential fragment ions at *m*/*z* 215 and 189 probably produced due to the cleavage of B ring. Based on these MS/MS data, peak **10** was identified as crinamabine which was reported from other plants in the past [34].

#### 2.2.6. Identification of Homolycorine Type Alkaloids

As for MS/MS data derived from homolycorine type alkaloids, major fragment ions were observed due to RDA cleavage and the losses of some substituents. Peaks **7**, **19**, **23**, **26**, **27**, **28**, **30** and **36** were classified into homolycorine type group and identified based on their MS/MS data.

**Figure 4:**
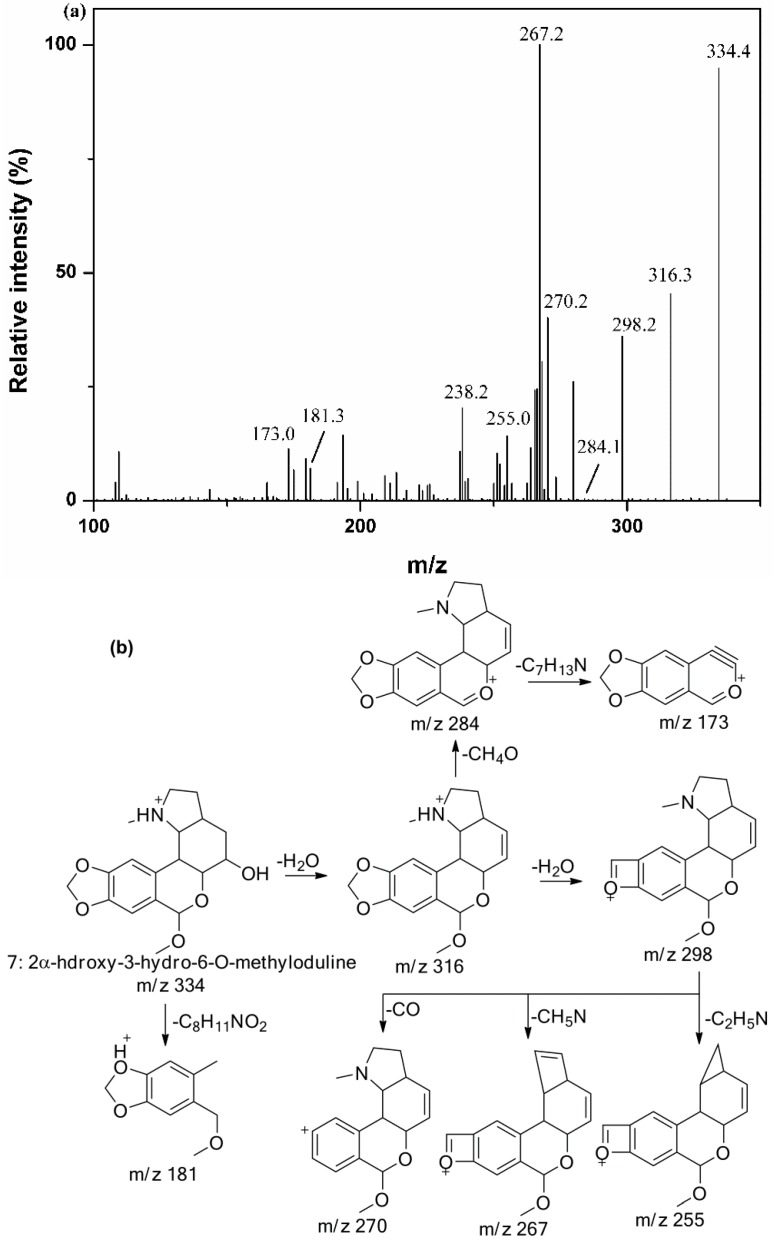
The MS/MS spectrum of peak 7 (**a**) and the proposed fragmentation pathways (**b**).

The MS/MS spectrum of peak 7 with the [M + H]^+^ ion at *m*/*z* 334 was shown in Figure 4a, and it could be tentatively identified as 2α-Hydroxy-3-hydro-6-*O*-methyloduline [35] based on the proposed fragment pathways in Figure 4b.

Peaks **19**, **26**, **27** and **36**, which showed the corresponding [M + H]^+^ ions at *m*/*z* 302, 316, 332 and 346, respectively, were identified as oduline, hippeastrine and 2α-Hydroxy-6-*O*-methyloduline, 2α-Methoxy-6-*O*-methyloduline by comparing their MS/MS spectra with those reported standards [35]. In the MS/MS spectrum of peak **23**, fragment ions at *m*/*z* 275, 267, 255, 243 and 223 were produced mainly due to the loss of N vicinal moiety, the fragment ion at *m*/*z* 195 was observed because of the further loss of CO from the fragment ion at *m*/*z* 223, and another fragment ion at *m*/*z* 124 was produced by RDA cleavage. Thus, peak 23 was identified as hippeastrine N-oxide [36].

In regard to peak **28**, the abundant fragment ion at *m/z* 207 was produced by RDA cleavage from its [M + H]^+^ ion at *m*/*z* 316. Comparing the MS/MS data with those of peak **26**, the fragment ion at *m*/*z* 126 was not observed, suggesting the lack of a hydroxyl at C-2. Thus, peak **28** was tentatively identified as (+)-8,9-methylenedioxyl-homolycorine N-oxide [5]. For peak **30**, the fragment ion at *m*/*z* 300 corresponding to the loss of methyl group from its parent ion at *m*/*z* 316 [M + H]^+^ , and another fragment ion at *m/z* 191 was observed due to the loss of C_8_H_15_NO (141 Da) produced by RDA cleavage. Therefore, peak **30** was tentatively identified as 2-methoxyoduline [35].

#### 2.2.7. Identification of Tazettine Type Alkaloids

**Figure 5:**
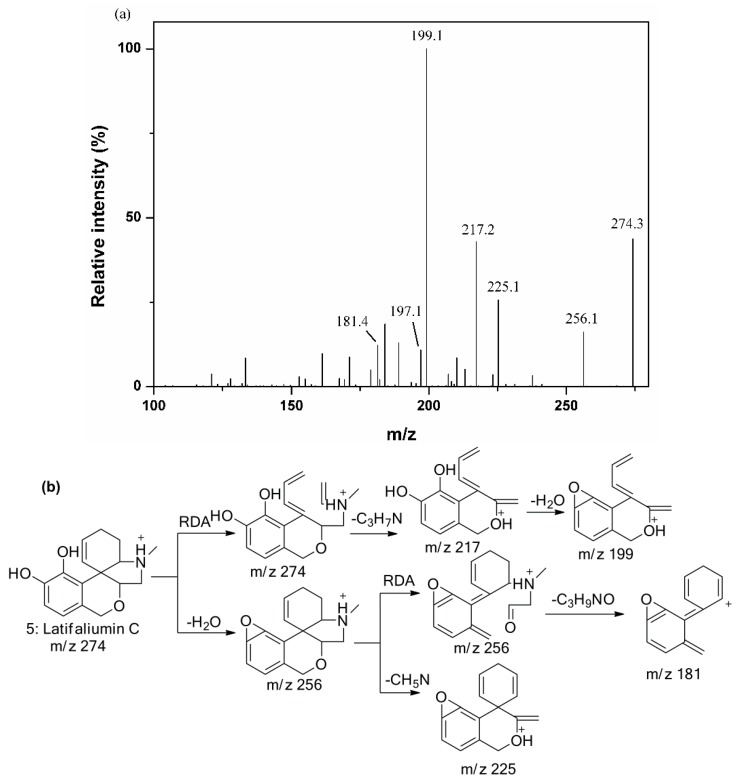
The MS/MS spectrum of peak 5 (**a**) and the proposed fragmentation pathways (**b**).

According to their MS/MS data and the proposed fragmentation patterns, peaks **3**, **4** and **5** could be classified as tazettine type alkaloids. The MS/MS spectrum and the proposed fragment pathway of peak **5** were shown in Figure 5. In the MS/MS spectra of peak **3** and **5**, the same series of fragment ions at *m*/*z* 225, 217, 199 and 181 were detected, and the neutral losses of H_2_O from the corresponding [M + H]^+^ ions at *m*/*z* 290 and 274 produced fragment ions at *m*/*z* 272 for peak **3** and *m*/*z* 256 for peak **5**, respectively. By comparing their MS/MS spectra with those of the authentic standards reported, peak **3** was identified as 3-hydroxylatifaliumin C, while peak **5** was identified as Latifaliumin C [33]. For peak **4**, the major fragment ions at *m*/*z* 258, 219, 201 and 173 were 2 Da more than those corresponding fragment ions at *m*/*z* 256, 217, 199 and 171 of peak **5**. In addition, the molecular ion at *m*/*z* 276 of peak **4** also showed 2 Da more than that of peak **5** and was tentatively identified as dihydro-latifaliumin C [33].

### 2.3. Comparison of AAs among the Three Lycoris Species

**Figure 6:**
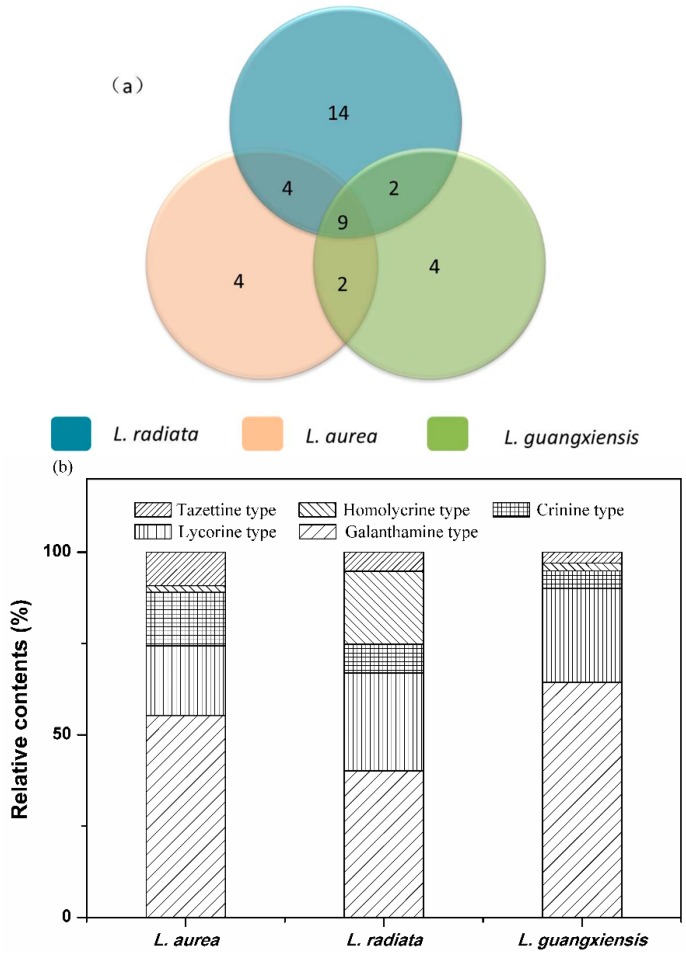
The number of AAs (**a**) and relative contents of five types of AAs (**b**) detected from *L. radiata*, *L. aurea* and *L. guangxiensis*.

Based on the identification of AAs from the three Lycoris species above, a comparison of AAs among different species was conducted, which was depicted in Figure 6a. It can be summarized that 19 alkaloids were found in the sample of *L. aurea*, while 28 and 18 in *L. radiata* and *L. guangxiensis*, respectively. Of these, nine AAs (peaks numbered with **4**, **8**, **11**, **13**, **14**, **17**, **19**, **21** and **22**) are the common components existing in all three species, and some components were found only in two of them, which are four AAs (peaks numbered as **2**, **15**, **25**, and **26**) in *L. radiata* and *L. aurea*, two AAs (peaks numbered as **3** and **10**) in *L. radiata* and *L. guangxiensis*, and two AAs (peaks numbered as **9** and **24**) in *L. aurea* and *L. guangxiensis*, respectively. To find potential AAs markers for the identification or quality control of different species from genus *Lycoris*, unique peaks of these three species were successfully picked out, which are four AAs (peaks numbered with **5**, **33**, **37**, and **38**) in *L. aurea*, 14 AAs (peaks numbered as **1**, **6**, **7**, **12**, **20**, **23**, **27**, **28**, **29**, **30**, **32**, **34**, **35**, and **39**) in *L. radiata*, and four AAs (peaks numbered as **16**, **18**, **31**, and **36**) in *guangxiensis*, respectively. Briefly, the crosscutting and inclusion relationships of AAs found in these three *Lycoris* species can provide great knowledge on both quality control for these species and better understanding of the correlation between AAs and their associated activities.

Since the evaluation of bioactivity of AAs in one plant species depends on both the qualitative and quantitative knowledge, the quantitative information of the five types of AAs in the three Lycoris species was then investigated based on their LC chromatograms, which was shown in Figure 6b. It can be seen that galanthamine and lycorine type alkaloids were the two major types of AAs accounting for 55.3% and 19.1% of galanthamine and lycorine type in *L. aurea*, 40.1% and 26.9% of galanthamine and lycorine type in *L. radiate* and 64.4% and 25.6% of galanthamine and lycorine type in *L. guangxiensis*, respectively. However, the discrepancies were mainly found in the contents of the other three types among the three species. In *L. aurea*, those three types account for about 25.6% from the total AAs, which are 1.8%, 14.6%, and 9.2% for homolylcorine, crinine, and tazettine types of alkaloids, respectively. In *L. radiata*, the homolylcorine type is another remarkable type making up 20.0%, which is next to the lycorine type, followed by the crinine type with 7.8% and tazettine with 5.2%, respectively. While in *L. guangxiensis*, the remainig three types together account for only 10.0% of the total AAs, in which 4.9% constitutes the crinine type, followed by 3.0% for the tazettine type and 2.1% for the homolylcorine type, respectively.

### 2.4. Comparison of Anti-HepG2 Activity of AAs in the Three Lycoris Species

**Figure 7:**
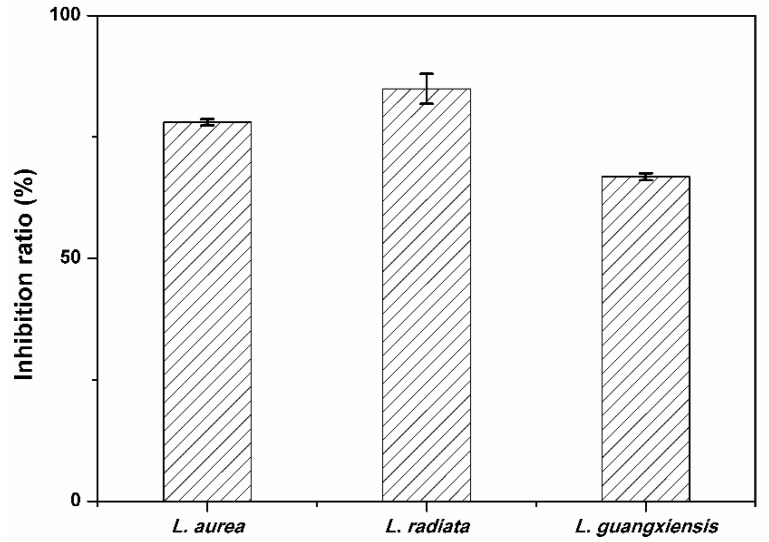
The anti-HepG2 activity of AAs from *L. aurea*, *L. radiata* and *L. guangxiensis*. (The error bars indicated RSDs calculated from triplicate tests).

AAs were the major anti-tumor compounds from genus *Lycoris* plants. Undoubtedly, the discrepancies of AAs composition and contents in different *Lycoris* species will result in differences in their bioactivity. Therefore, a comparison of the anti-HepG2 activity of the total AAs extracted from the three *Lycoris* species was conducted, in which CCK-8 (Cell Counting Kit-8) was employed. The result is shown in Figure 7. It was observed that the three species showed different inhibitory rates of HepG2 at 78.02%, 84.91% and 66.81% for *L. aurea*, *L. radiate* and *L. guangxiensis*, respectively, indicating that there exist differences in bioactivity of AAs from the three species. However, the structural subgroups and anti-cancer investigation indicated that homolycorine type (nobilisitine B and clivonine) and tazettine type (tazettine) AAs displayed no notable activity, while some of the lycorine and crinine type AAs showed significant cytostatic effects on actin cytoskeleton rigidity [37]. Nevertheless, pretazettine belonging to the tazettine type was reported to inhibit the growth of Hela cells [12]. (+)-8-hydroxy-homolycorine-α-N-oxide belonging to homolycorine type exhibited significant cytotoxicity against the tested seven tumor cell lines [38]. Considering the multi-component AAs and their distinct bio-activities, the anti-HepG2 activities could be attributed to the synergistic effects of the samples tested.

To determine the potential of anti-HepG2 activities of different types of AAs, a preliminary investigation into the correlations between the inhibitory rates and the content of AAs among the three *Lycoris* species was conducted. It can be observed from Figure 6b that the two remarkable types of AAs, *i.e.*, galanthamine and lycorine type alkaloids, may result in the general anti-HepG2 activity, with inhibitory rates ranging from 66.81%–84.91% among the three species. The highest inhibitory rate (*i.e.*, 84.91%) observed from *L. radiata* can be attributed to its remarkable content of homolylcorine type alkaloids which were found to be much lower in the other two species. Thus, in this study, galanthamine, lycorine, and homolylcorine type alkaloids were proposed as the most promising anti-HepG2 AAs from the *Lycoris* species. This may contribute to the further identification of the anti-tumor components in the AAs from *Lycoris* species in the future.

## 3. Experimental Section

### 3.1. Chemicals

Formic acid, ammonium acetate (AA) and acetonitrile (ACN) of HPLC grade were purchased from ROE Scientific INC, ANROUR Chemicals Supply, and Fisher Scientific, respectively. Oasis MCX cartridges (500 mg/3 mL) used for sample preparation were purchased from Weltch CO. (Wuhan, China). Methanol, Ethanol, Chloroform, Hydrochloric acid (HCl), Petroleum ether (PE), ammonia, and acetone were all of analytical grade and purchased from Sino-pharm chemical Reagent CO. Water for HPLC and LC-MS was prepared with EPED (Nanjing Yeap Esselte Technology Development Co., Nanjing, China).

### 3.2. Plant Materials and Sample Preparation

Identified to be *Lycoris radiata* (*L. radiata*), *Lycoris aurea* (*L. aurea*) and *Lycoris guangxiensis* (*L. guangxiensis*), fresh *lycoris* materials of three species were collected from Wuhan botanical garden in April 2014. The bulbs of *lycoris* were cut into slices and dried in the oven below 30 °C. The dry slices were weighed, and ultrasonically extracted with 90% ethanol for 30 min. After repeated extraction three times, the extracts were combined and filtered, then the supernatants were evaporated under reduced pressure to afford syrup residues. The resulted residues were dispersed in 5% HCl, and extracted with petrol ether (PE) to remove chlorophyll. The pH value of water phase was adjusted to 9.5 with ammonia solution, and further extracted repeatedly with chloroform. The chloroform phase was combined and concentrated to yield crude AAs. An aliquot of crude AAs solutions was further enriched with Oasis MCX (500 mL/3 mL) cartridges in the following steps: firstly, the cartridges were activated with methanol (3 mL) and methanol–water (85:15, 3 mL); secondly, samples dissolved in methanol with 1% formic acid solution were loaded to the cartridges, and methanol was used as wash solution; thirdly, the AAs for analysis were eluted with 3 mL of 5% ammonia in acetone. At last, the eluents were collected and dried with Pressure Blowing Concentrator. The residues were dissolved with 5% methanol, filtered with 0.22 micro-filter membrane and stored at 4 °C before HPLC-UV and LC-MS analysis.

### 3.3. HPLC-UV/ESI-MS/MS Analysis

#### 3.3.1. HPLC-UV Conditions

A Thermo Accela 1250 HPLC consisting of an auto-sampler coupled with a UV-visible detector (Thermo Fisher Scientific, San Jose, CA, USA) was employed for the analysis of AAs. A 10 μL aliquot of AAs solution was injected and analyzed on a Phenomenex ODS column (150 × 2.00 mm, 5 μm, Phenomenex). The column temperature was set at 30 °C, and the flow rate was 0.2 mL/min. The mobile phase A and B were 40 mM ammonium acetate aqueous solution and acetonitrile, respectively. The gradient was set as follows: 0–15 min, 5% (B); 15–17 min, 5%–10% (B); 17–20 min, 10% (B); 20–30 min, 10%–18% (B); 30–55 min, 18%–68% (B). The chromatogram was recorded at a wavelength of 232 nm.

#### 3.3.2. ESI-MS/MS Conditions

For the ESI-MS/MS experiment, a Thermo Accela 600 HPLC system with a UV detector coupled to a TSQ Quantum Access MAX mass spectrometer (Thermo Fisher Scientific, San Jose, CA, USA) was used for the LC-MS analysis in the positive mode. MS conditions were set as follows: mass range from 200–1000 Da; Spray Voltage, 3.0 kV; Capillary temperature, 250 °C; Sheath gas pressure, 40 psi; Aux gar pressure, 10 psi.

### 3.4. Quantitative Analysis of AAs

The relative contents of AAs were calculated based on the peak areas from the LC chromatography, and the identification of the corresponding LC peaks was conducted by LC-MS/MS and compared with references or some standard AAs.

### 3.5. Anti-HepG2 Activity Test

The anti-HepG2 activity of the crude extracts was tested using human hepatic carcinoma cell line (HepG2, from CCTCC (China Center for Type Culture Collection)) with Cell Counting Kit-8 (CCK-8). Cells were cultured in a 96-well plate at a density of 5000 cells per well in DMEM (Dulbecco’s Modified Eagle Medium) supplemented with 10% fetal bovine serum (FBS). After cultured under 5% CO_2_ at 37 °C for 24 h, the cells were treated with AAs at the concentrations of 10 μg/mL in 3 duplicates. DMSO was used as control. After incubation for 48 h, 10 μL of CCK-8 was added to each well. Another 2 h later, the optical density (OD) values were determined at 450 nm by microliter plate reader (MIOS Junior, Merck, Hercules, CA, USA). The inhibitory rate (%) equals to (ODC − ODT)/ODC × 100% [39,40], here, ODT and ODC were the OD values of blank control and alkaloids extracts, respectively.

## 4. Conclusions

In this study, an HPLC-UV/ESI-MS/MS method was employed to analyze and compare the fingerprint profiles of AAs from *L. aurea*, *L. radiata* and *L. guangxiensis*. As a result, 39 peaks corresponding to AAs were detected and 29 of them were identified by comparing their LC-MS/MS spectra with the corresponding standards reported. Twelve peaks of the identified AAs were firstly observed in *Lycoris* genus, of which five peaks belonged to the Amaryllidaceae family. To distinguish *lycoris* species from other plants rich in AAs, peaks **11**, **13** and **14** corresponding to lycorine, lycoramine and galanthamine together could be selected as the marker of genus *Lycoris* in the Amaryllidaceae family. For these three species, peaks **5**, **7** and **31** corresponding to Latifaliumin C in *L. aurea*, 2α-Hydroxy-3-hydro-6-*O*-methyloduline in *L. radiata* and 3,11-dimethoxy-lycoramine in *L. guangxiensis* were the unique peaks which could be selected as the AAs marker for these three species, respectively. It is further revealed for the first time that the three species under investigation were different not only in the types of AAs, but also in their contents, and both contributed to their pharmacological distinctions. To the best of our knowledge, the current research provides the most detailed phytochemical profiles of AAs in these species, and offers valuable information for future valuation and exploitation of these medicinal plants.
